# The sneeze reflex in physiological and pathological states: a mini review

**DOI:** 10.3389/fnins.2025.1598027

**Published:** 2025-05-09

**Authors:** Yang Rui, Tianyuan Xin, Yu Chen, Beiyi Xiang, Changwen Chen, Rong Dong, Zhe Chen

**Affiliations:** ^1^Laboratory of Cough, Kunshan Key Laboratory of Chronic Cough, Affiliated Kunshan Hospital of Jiangsu University, Suzhou, China; ^2^Department of Physiology and Pharmacology, Southeast University, Nanjing, China

**Keywords:** sneeze reflex, innervation, central regulation, pathological manifestations, cough

## Abstract

The sneeze reflex serves as a protective response of the human body to environmental stimuli, with its regulatory mechanism largely depending on a complex nervous system. Current research indicates that the sneeze reflex can be triggered by both internal and external stimuli, including light and temperature. The composition of its reflex arc involves the coordinated action of multiple neural structures. Recent in-depth studies on the mechanism of the sneeze reflex have revealed a growing association between this reflex and various diseases, such as allergic rhinitis and infectious diseases. However, existing research primarily focuses on elucidating the neurophysiological basis of the sneeze reflex and its related neural pathways. The manifestations and mechanisms of the sneeze reflex under different pathological states remain underexplored, thereby limiting the potential application of the sneeze reflex in clinical diagnosis and treatment. This review aims to discuss the physiological mechanisms and pathological manifestations associated with the sneeze reflex.

## Introduction

The sneeze reflex is a protective physiological response of the body, designed to rapidly expel irritants or pathogens from the respiratory tract. Although this reflex is a common occurrence in daily life, it involves intricate neural pathways and the release of neurotransmitters. Recent studies have clarified that the neural circuit governing the sneeze reflex necessitates the close cooperation of several brain regions, particularly the sneeze-inducing area of the trigeminal nucleus located in the medial ventral part of the spinal cord and the central sensory area of nasal sensory neurons (Li et al., 2021; Nonaka et al., 1990; Seijo-Martínez et al., 2006). Research has demonstrated that neuromodulin B (NMB) plays a crucial role in the transmission of the sneeze reflex; the absence of NMB receptors or damage to related neurons results in the loss of this reflex (Li et al., 2021). Furthermore, the central mechanism regulating the sneezing reflex is closely associated with the immune response, as immune cells can influence neuronal activity by releasing cytokines and neuropeptides, thereby modulating the sneezing reflex (Kabata and Artis, 2019; Voisin et al., 2017).

The sneeze reflex is often closely associated with conditions such as allergic rhinitis and viral respiratory infections. In the context of respiratory infections, the primary mode of transmission occurs through aerosolized droplets containing viruses, which are expelled during sneezing or coughing (Bourouiba, 2020; Tang et al., 2006). Notably, a single sneeze can release up to 40,000 virus-laden droplets. These droplets can travel through the air for distances of 7–8 meters and remain suspended for up to 10 min, thereby heightening the risk of virus transmission (Bourouiba, 2016; Bourouiba, 2020; Scharfman et al., 2016). Furthermore, individuals exhibit varying sensitivities to the sneezing reflex. Some individuals may exhibit a sneezing response when exposed to strong light. This phenomenon is referred to as the “photo-induced sneeze reflex” (Shetty et al., 2023). The precise mechanism underlying this reflex remains unclear, but it may be associated with corneal nerve reflexes or prolonged smoking habits (Kulas et al., 2017; Sevillano et al., 2016).

By studying the mechanisms of the sneeze reflex and its manifestations in various pathological states, we can develop more effective diagnostic and treatment methods for clinical practice, thereby enhancing the quality of life for patients. This review will summarize the triggering factors, neural-central mechanisms and pathological manifestations of the sneeze reflex, providing references for further research and clinical application.

### Basic concepts and physiological significance of the sneeze reflex

The sneeze reflex is primarily triggered by the stimulation of receptors located in the mucous membranes of the nasal cavity and throat. These receptors can detect external stimuli such as pollen, smoke, and other particulate matter. In the nasal region, receptors receive signals from these stimuli and initiate the sneezing response via incoming C-type neurons and cholinergic neurons (Baraniuk and Kim, 2007). Although this process appears to be a simple physiological response, it actually involves the integration and regulation of the more complex central nervous system.

According to the latest research data, the airflow speed generated during sneezing can reach nearly 100 kilometers per hour (Han et al., 2021). When the receptors in the nasal cavity are stimulated, the sneeze reflex is quickly initiated, and through this strong airflow, foreign particles and irritants are expelled from the body (Burke, 2012). This process not only helps to remove harmful substances from the nasal cavity but also prevents these substances from entering the lower respiratory tract, thereby reducing the risk of infection. The occurrence of the sneeze reflex can be influenced by various factors; however, in some infectious diseases, the primary cause is the presence of inflammatory reactions. These inflammatory reactions directly stimulate the receptors in the body, triggering a strong reflexive sneeze that causes air to burst out rapidly (Singh et al., 2021). Therefore, sneezing is not only a response to local stimulation but is also associated with the body’s overall immune response, aiding in the management of infections and inflammation.

### Triggers of the sneeze reflex

The sneeze reflex primarily serves to expel foreign substances or irritants from the respiratory tract. Common external stimuli include odorants, dust, pollen, and smoke. When the nasal cavity comes into contact with irritating odorant molecules, such as capsaicin and ammonia, it primarily stimulates the trigeminal nerve, leading to sensations such as stinging, cooling, burning, or pain, which can induce sneezing. In contrast, exposure to non-irritating odorants and their qualities, including type and familiarity, primarily stimulates the olfactory nerve to trigger the sneeze reflex (Badran et al., 2022). Although their roles in odorant perception differ, they complement each other. Moreover, most odorants, including the “smoky” guaiacol (GUA), which possesses strong trigeminal properties, are capable of activating both the olfactory and trigeminal nerves at the same time. However, certain odorants exhibit relatively weak trigeminal properties. For instance, phenylethyl alcohol (PEA), an odorant that evokes a “rose-like” scent and is frequently used in clinical evaluations of olfactory function, is regarded as one of the “olfactory” odorants (Doty et al., 1978). Microorganisms, bacteria, and fungal spores present in dust may also act as triggers for sneezing, particularly in humid or polluted environments (Eriksen et al., 2023; Gabriel et al., 2016). Moreover, internal stimuli are typically the body’s natural responses to potential threats. Common internal stimulus factors include various infections, such as viral or bacterial infections, as well as inflammatory responses within the body. An allergic reaction is an internal irritation triggered by specific allergens, such as dust mites and animal dander. Upon entering the body, these allergens can directly damage respiratory epithelial cells, subsequently activating mast cells to release chemical substances like histamine, which results in inflammatory reactions and triggers the sneeze reflex (Calderón et al., 2015).

Changes in light and temperature can also trigger the sneeze reflex. Light stimulation, particularly intense light (primarily strong sunlight), has been confirmed to elicit a specific sneeze reflex known as the light-induced sneeze reflex. This condition is inherited in an autosomal dominant manner and is relatively common among certain individuals (Hakim and Alsaeid, 2019). The precise cause of this condition remains incompletely understood. It may be attributed to individuals exhibiting heightened sensitivity to visual stimuli processed by the visual cortex, accompanied by activation of the somatosensory area (Langer et al., 2010). Furthermore, the sudden exposure to cold or hot air may also stimulate nasal receptors, leading to the occurrence of the sneeze reflex (Tamura, 2011; Yonghua et al., 2022). These atypical triggering factors underscore the complexity of the sneeze reflex mechanism, which may involve the interaction of multiple physiological and environmental factors.

In addition to the aforementioned factors, certain medications can also decrease the frequency of sneezing by inhibiting the inflammatory response within the nasal cavity. For instance, antihistamines and analgesics have been noted in this regard (Hakim and Alsaeid, 2019). Consequently, when administering local anesthesia, the inclusion of fentanyl may help to lessen the incidence of sneezing (Parker et al., 2023). However, it is important to note that the side effects of certain medications can also trigger the sneeze reflex; for example, non-steroidal anti-inflammatory drugs (NSAIDs) (Bindu et al., 2020). The influence of medications on the sneeze reflex not only encompasses their therapeutic effects but is also related to individual differences and the specific conditions of patients.

### Innervation of the sneeze reflex

**FIGURE 1:**
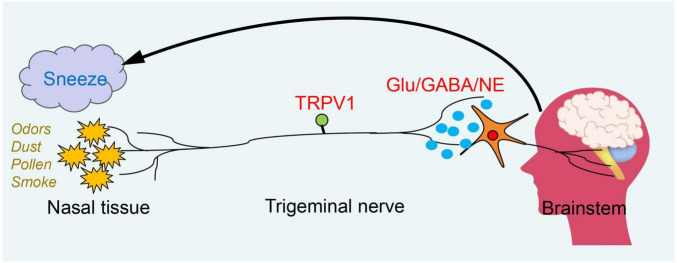
The neural mechanism of the sneeze reflex. The urge to sneeze is initiated when an external irritant stimulates sensory nerve endings in the nasal passages. The stimulus signal is transmitted through the central axon to the brainstem, where it activates the sneezing reflex. This process is dependent on the action of neurotransmitters.

The sneeze reflex is primarily initiated by chemical or mechanical stimulation of the nasal or nasopharyngeal mucosa, predominantly relying on the primary afferent fibers of the trigeminal nerve (Figure 1). Current experiments have confirmed that in mice with MrgprC11+ neurons knocked out, the sneezing response is nearly eliminated when exposed to nebulized histamine, serotonin, and capsaicin (Jiang et al., 2024). The MrgprC11+ sensory neurons within the nasal cavity have been identified as “sneeze neurons,” which specifically express NMB and can respond to stimuli such as capsaicin, histamine, allergens, and the influenza virus. These neurons activate NMBR+ neurons in the brainstem’s sneeze-evoking region by releasing NMB (Jiang et al., 2024; Li et al., 2021). In the sneeze reflex, TRPV1 and NMB are co-expressed in nasal neurons. Some studies have shown that TRPV1 is a cation channel sensitive to capsaicin and serves as a downstream transduction channel of the histamine H1R receptor (Plant et al., 2007; Shim et al., 2007). By administering a resiniferatoxin (RTX) solution into the nasal cavity of mice to ablate TRPV1 nasal sensory fibers, we observed a significant reduction in the sneezing response to both capsaicin and histamine (Li et al., 2021). This finding indicates that TRPV1 is also involved in the signal transduction of chemical stimuli. Utilizing the S1 protein to stimulate airway receptors in mice and combining calcium ion imaging with electrophysiological recordings, researchers observed the activation of C fibers (Kim et al., 2023). The study found that the S1 protein can directly activate sensory C fibers in the respiratory tract, thereby triggering responses such as sneezing. The realization of the sneeze reflex not only depends on the activation of nasal sensory nerves but also requires the involvement of motor nerves. When the sensory nerves in the nasal cavity are activated, the central nervous system quickly and efficiently sends signals to direct the motor nerves to activate the relevant muscle groups, thereby effectively promoting the occurrence of the sneeze reflex. Moreover, the neural regulation of the sneeze reflex requires not only the participation of both sensory and motor neurons but also the integration and coordination of complex neural circuits. Neurotransmitters play a crucial role in this process, including glutamate, γ-aminobutyric acid, and monoamine neurotransmitters. For instance, glutamate enhances the excitability of the trigeminal nucleus by activating N-methyl-D-aspartate (NMDA) receptors, thereby promoting signal transmission to the SEZ (Karsan et al., 2025).

### Regulation of the central nervous system

The medulla oblongata, serving as the central integration point for the sneeze reflex, is situated between the pons and the spinal cord. It is responsible for transmitting sensory, motor, and autonomic nervous system information (Nieuwenhuys, 2011). The highly conserved structure of the medulla oblongata contributes to its evolutionary stability and contains multiple nuclei, thereby ensuring the coordination and normal functioning of all bodily functions (Diek et al., 2022). Among these, the sneeze reflex primarily involves two key nuclei: the sneeze-evoking zone (SEZ) and the caudal ventral respiratory group (cVRG). SEZ anatomically corresponds to the central receptor area of the nasal sensory neurons, which primarily integrates various sensory inputs from the nasal cavity to direct the sneeze reflex, thereby triggering this physiological response (Li et al., 2021; Nonaka et al., 1990). Specifically, olfactory receptors may interact with the intracellular cAMP signaling pathway via G protein-coupled receptors, resulting in the generation of electrical signals. These signals are transmitted to the olfactory bulb and other related brain regions through the olfactory nerve, ultimately activating the sneeze reflex within the central nervous system (Boccaccio et al., 2021). The SEZ is located on the dorsal side of the medulla oblongata and serves as the primary central integration node for afferent signals from the trigeminal nerve. Capsaicin, histamine, and other irritants activate TRPV1 receptors in the nasal cavity, prompting MrgprC11+ neurons to release NMB and transmit signals to the cVRG via synaptic connections. Knocking out the NMBR gene in mice or administering NMBR antagonists can significantly inhibit the sneeze reflex. The cVRG, located in the ventral medulla, is responsible for coordinating the physiological actions associated with sneezing. Upon receiving the signal, the cVRG activates the respiratory muscle groups and the soft palate muscles, thereby initiating the three stages of the sneeze reflex. The process begins with the inhalation phase, during which the diaphragm and intercostal muscles contract to draw in a large volume of air. This is followed by the compression phase, wherein the soft palate muscles contract, causing the uvula to descend and seal the nasopharyngeal passage. Finally, the explosive exhalation phase occurs, during which the glottis opens, allowing high-speed airflow to be expelled through the nasal cavity (Sangha et al., 2022).

### The difference from the cough reflex

The sneeze reflex and the cough reflex are both protective mechanisms of the respiratory tract; however, they exhibit significant differences in their triggering mechanisms and neural pathways. The cough reflex originates from the larynx and lower respiratory tract (such as the trachea and bronchi), mediated by the vagus nerve. The peripheral vagus nervous system that governs the respiratory tract primarily comprises Aδ fibers and C fibers. Among these, mechanical stimuli in the throat (such as foreign objects) are transmitted through Aδ fibers, while chemical stimuli (such as gastric acid reflux) depend on TRPV1 and P2X3 receptors located on C fibers (Canning, 2010). In a mouse cough model, selective activation of somatostatin (SST+) neurons innervating the trachea using Ly344864 and IL-31 significantly increased the frequency of coughing compared to the control group (Jiang et al., 2024). These types of neurons are sensitive to chemical stimuli such as ammonia and bradykinin, and the signals are directly transmitted to SST-sensitive neurons in the nucleus tractus solitarius (NTS) of the brainstem rather than through the NMBR+ pathway. In the cough reflex, the laryngeal POU class 2 homeobox 3 (Pou2F3) chemosensory cells communicate with the vagus nerve through the calcium homeostasis modulator 3 (CALHM3) channel synapse, thereby triggering purinergic signaling, such as ATP release (Soma et al., 2025). During the central integration process, the cough reflex primarily depends on the medullary respiratory center to coordinate the inspiratory and expiratory phases, projecting to the cVRG through prodynorphin-expressing (Pdyn+) NTS neurons, thereby forming the Pdyn+ NTS-cVRG circuit that regulates the rhythmic actions of coughing (Lu et al., 2024). By establishing a mouse cough behavioral model and integrating single-cell sequencing technology, this study revealed that tachykinin 1 (Tac1) neurons are a critical component of the airway-vagal-brain neural circuit responsible for regulating the defensive cough response in mice (Gannot et al., 2024). In the motor output process, the cough reflex predominantly relies on the function of the phrenic nerve and intercostal nerves, generating a high-speed oral airflow through the rapid closure and subsequent sudden opening of the glottis (Polverino et al., 2012).

### Allergic rhinitis

**FIGURE 2:**
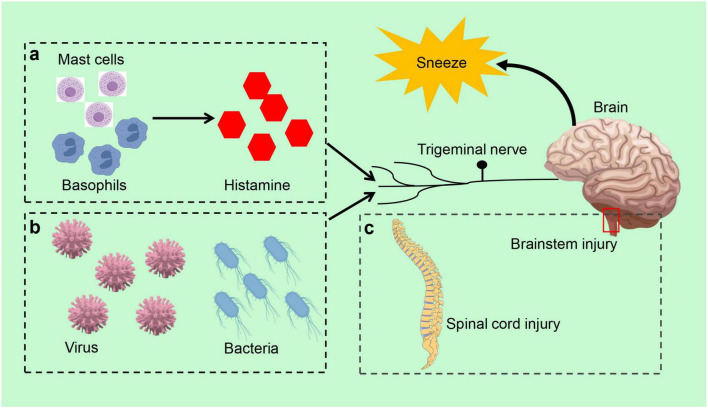
Sneeze reflex related disorders. **(a)** In allergic rhinitis, mast cells and basophils initiate the sneeze reflex by releasing inflammatory mediators, such as histamine, which activate sensory neurons in the nasal cavity. **(b)** Infectious diseases, the sneeze reflex is typically triggered by infections caused by viruses and bacteria. **(c)** Brainstem or spinal cord injury can also elicit the sneeze reflex.

Allergic rhinitis is a prevalent IgE-mediated chronic inflammatory disease characterized by frequent sneezing, runny nose, itchy nose, and nasal congestion (Bernstein et al., 2024; Varshney and Varshney, 2015). Neuroimmune interactions significantly influence the severity and duration of allergic symptoms (Cong et al., 2024; Klimek et al., 2023; Konstantinou et al., 2022). In allergic rhinitis, histamine and other inflammatory mediators released by mast cells and basophils stimulate sensory neurons in the nasal cavity. These neurons transmit signals to the sneezing center in the brainstem via neural pathways, altering the processing of the central nervous system and affecting sympathetic, parasympathetic, and autonomic nerve transmission in the intestine, ultimately triggering the sneezing reflex (Konstantinou et al., 2022; Undem and Taylor-Clark, 2014; Figure 2a). Compared to non-allergic rhinitis patients, individuals with allergic rhinitis typically exhibit a higher frequency and intensity of the sneeze reflex (Ríos-Deidán et al., 2023). Additionally, allergic rhinitis may lead to ear complications, further impacting patients’ quality of life, such as recurrent secretory otitis media (Kwon et al., 2013; Tukur et al., 2022). In clinical practice, physicians can initially ascertain the presence of allergic rhinitis by observing the patient’s sneeze reflex. They can also assess the severity of the disease based on the intensity and frequency of this reflex. For instance, in allergen challenge tests, a rapid onset of the sneeze reflex, combined with a significant increase in the number of sneezes following exposure to allergens, can be utilized to confirm the diagnosis of allergic rhinitis.

### Infectious diseases

The sneeze reflex is closely associated with infectious diseases and is typically triggered by pathogens such as viruses or bacteria (Figure 2b). A viral infection induces an inflammatory response in the upper respiratory tract, stimulating the sensory neurons in the nasal cavity, which subsequently triggers sneezing. Infectious diseases can also influence the strength and frequency of the sneeze reflex by altering nervous system function (Zaccone and Undem, 2016). The sneeze reflex serves not only as a defense mechanism to expel pathogens but also as an immune response to infection (Li et al., 2021). In the context of respiratory infections, the speed of airflow generated by sneezing and the size of aerosolized particles can significantly impact the extent of virus transmission, thereby increasing the risk of infection (Joseph et al., 2022). For instance, sneezing was identified as one of the primary modes of transmission for the novel coronavirus during its epidemic (Hawks et al., 2021; Rabaan et al., 2021). Consequently, in response to public health events, the transmissible nature of sneezes motivates individuals to adopt protective measures, such as wearing masks and maintaining social distance, to minimize the risk of infection associated with sneezing (Kalin Ünüvar et al., 2021).

Sinusitis is a condition characterized by infection or inflammation of the sinuses, typically classified into two types: acute and chronic (Leung and Katial, 2008). Acute sinusitis is usually precipitated by an upper respiratory tract infection, with common pathogens including influenza viruses, adenoviruses, and bacteria (DeMuri et al., 2019; Isaacson, 2015). In contrast, chronic sinusitis is defined by symptoms persisting for more than 12 weeks, often accompanied by the formation of nasal polyps (Kwah and Peters, 2019). The pathogenesis of chronic sinusitis is complex and may involve an abnormal immune response and dysregulation of the microbial community (Shah and Kobayashi, 2023). Patients with chronic sinusitis frequently exhibit eosinophilic infiltrates, suggesting a potential association with allergic reactions (Fokkens et al., 2019; Tokunaga et al., 2015). Individuals suffering from sinusitis often experience frequent sneezing, primarily due to an inflammatory response in the nasal passages and sinuses. The frequency and intensity of sneezing may correlate with the severity of sinusitis, with those experiencing severe cases demonstrating more frequent and intense sneezing responses. This reaction not only impacts the patient’s quality of life but can also lead to complications such as orbital infections or sinus bronchial allergic fungal disease (Boiko et al., 2023; Bozeman et al., 2011; Carr, 2016).

### Neurologic disorders

The generation of the sneeze reflex relies on the integrity of the central nervous system, particularly the proper functioning of the brainstem in critical regions of the body (Li et al., 2021; Figure 2c). In cases of brainstem or spinal cord injury, there is an increased threshold for the sneeze reflex, which may manifest as a weakening or absence of the reflex. Abnormalities in the sneeze reflex are also strongly associated with various neurological disorders, such as Parkinson’s disease and multiple sclerosis. Multiple sclerosis is characterized as an inflammatory demyelinating disease of the central nervous system, while Parkinson’s disease is a progressive neurodegenerative disorder marked by tremor and bradykinesia (Hayes, 2019; Wootla et al., 2012). These disorders typically accompany impairment of neurological function, which in turn affects the regulation and management of the sneeze reflex by the central nervous system. Furthermore, neuro-immune interactions in central nervous system disorders may lead to dysregulation of the sneeze reflex, thereby affecting the body’s ability to respond to external stimuli (Yoshida et al., 2022). These effects extend beyond the sneeze reflex itself, potentially leading to abnormalities in other respiratory reflexes and impacting the patient’s overall respiratory function.

In clinical practice, the strength and frequency of the sneeze reflex serve as important indicators for assessing the functional status of the central nervous system. This reflex reflects the health and activity level of the nervous system and holds potential applications, particularly in the early diagnosis and monitoring of neurological disorders. By quantifying the characteristics of the sneeze reflex, physicians can more effectively evaluate a patient’s neurological status and tailor an individualized treatment plan. For instance, quantitative light reflex pupillometry (qLRP) has been proposed as a digital biomarker for assessing neurological function in neurodegenerative diseases such as Alzheimer’s disease (Gramkow et al., 2024). The reliability and validity of this assessment method introduce new perspectives for the clinical application of the sneeze reflex. Furthermore, the assessment of the sneeze reflex can be integrated with other neurological function tests to enhance the comprehensive evaluation of neurological disorders, such as video-based detection of blinking parameters for assessing idiopathic Parkinson’s disease (Jansen et al., 2024).

## Conclusion

The sneeze reflex is not merely a simple reflex action; rather, it is a complex process involving multiple neural pathways and regulated by various factors. In recent years, significant progress has been made in the research field concerning the neural mechanisms of the sneeze reflex, underscoring the important roles played by various neural pathways and neurotransmitters. Current research indicates that, while there are similarities in the studies of the sneeze reflex conducted by different scholars, significant differences exist in certain mechanisms and influencing factors. These differences may arise from variations in research methods, sample selection, and interpretations of physiological mechanisms. Therefore, future research should prioritize the standardization of research methods and the execution of multi-center, large-sample clinical trials to explore the physiological mechanisms and pathological functions of the sneeze reflex more comprehensively.

Currently, the majority of research primarily focuses on the neural mechanisms underlying the sneeze reflex, while studies examining its performance and variations across different populations—such as children, the elderly, or immunosuppressed patients—are relatively limited. Additionally, there are notable gaps in the investigation of how individual differences, including genetic factors, environmental influences, and underlying pathologies, affect the sneeze reflex. Future research should aim to further integrate bioinformatics, neurophysiology, immunology, and epidemiology to comprehensively elucidate the mechanisms of the sneeze reflex and its performance in various pathological conditions.

In conclusion, a comprehensive exploration of the neural central mechanisms underlying the sneeze reflex will aid in the identification and treatment of related diseases more effectively, ultimately enhancing the quality of life for patients. With the ongoing advancements in neuroscience and biomedical technology, studying the neural networks and molecular mechanisms associated with the sneeze reflex is becoming increasingly feasible, and this area is likely to emerge as a significant trend in future research.
